# Bacterial infection in severe acute pancreatitis patients admitted to the ICU

**DOI:** 10.1186/cc13388

**Published:** 2014-03-17

**Authors:** AR Prior, S Egan, R Durani, PG Murphy, J Febbell, G Fitzpatrick

**Affiliations:** 1 Tallaght Hospital, Dublin, Ireland

## Introduction

Controversy surrounds the empirical use of antibiotics in severe acute pancreatitis (SAP). There are concerns that the widespread use of antibiotic therapy in the absence of documented infection may lead to selection of drug-resistant organisms [1]. The aim of this study was to review the profile of pancreatic fluid isolates in patients with SAP admitted to the ICU.

## Methods

Data were reviewed for 38 patients admitted to the ICU over a 5-year period. We evaluated organisms cultured from pancreatic specimens, as well as the prevalence of drug-resistant organisms in this group of patients.

## Results

Aspirate of pancreatic material for culture was obtained in 55% of patients (*n *= 21). The mean time to acquisition of samples for culture from admission to ICU was 15.5 days. Fluid was sterile in 67% (*n *= 14) of initial samples. Gram-positive organisms were cultured from 43% (*n *= 9) of samples, Gram-negative organisms from 5% (*n *= 1) and yeasts from 5% (*n *= 1). Antibiotic therapy was administered in 95% of patients prior to samples being obtained for culture. On review of all samples received from patients (including nonpancreatic specimens), vancomycin-resistant enterococci (VRE) were isolated in 13 patients. Linezolid-resistant enterococci (LRE) were isolated in six patients, five of whom had VRE isolated prior to the culture of LRE. Extended- spectrum beta-lactamase organisms were isolated in two patients, and carbapenem nonsusceptible Gram-negative organisms in three patients. The mean APACHE II score was 18.5 and overall hospital mortality was 26%.

## Conclusion

In the majority of patients, initial aspirates of pancreatic material were sterile. This may be a result of prior antibiotic usage. Where organisms were cultured from initial aspirates, Gram-positive organisms predominated, possibly as a result of prior anti-Gram- negative antibiotic use. Therefore, in patients with ongoing sepsis who are receiving broad-spectrum antibiotic therapy, consideration needs to be given to the empiric treatment of Gram-positive infection, and in particular drug-resistant organisms such as vRe. Local epidemiology should be taken into account. Rationale use of antibiotics, in accordance with best-practice guidelines, may limit development of drug resistance; however, other risk factors for resistance may exist in this group and this would need to be further evaluated.
